# Surgical Excision of Duodenal/Pancreatic Metastatic Renal Cell Carcinoma

**DOI:** 10.3389/fonc.2014.00218

**Published:** 2014-08-14

**Authors:** Eduardo Espinoza, Ali Hassani, Ulka Vaishampayan, Dongping Shi, J. Edson Pontes, Donald W. Weaver

**Affiliations:** ^1^Surgical Oncology Division, Karmanos Cancer Institute, Detroit, MI, USA; ^2^Cayetano Heredia, Peruvian University, Lima, Peru; ^3^Medical Oncology Division, Karmanos Cancer Institute, Detroit, MI, USA; ^4^Department of Pathology, Karmanos Cancer Institute, Detroit, MI, USA; ^5^Department of Urology, Karmanos Cancer Institute, Detroit, MI, USA

**Keywords:** renal cell carcinoma, metastatic disease, renal surgery, nephrectomy

## Abstract

Renal cell carcinoma (RCC) has a potential to metastasize to almost any site and this may occur many years following nephrectomy. We present six cases with uncommon sites of metastasis: four patients presented with distal pancreatic metastasis and two with duodenal/head of the pancreas metastasis. Time to metastatic disease varied from 1 to 19 years following renal surgery. For patients are alive and two succumbed to their disease. Long-term survival can be achieved with aggressive surgical excision of disease.

## Introduction

Renal cell carcinoma (RCC) is the third most frequent malignancy of the genitourinary tract and it accounts for 3% of all adult malignancies (1). It has the highest mortality rate of the genitourinary cancers and its incidence has been rising steadily by 2–4% each year. If detected early, RCC is curable by excision although a minority is at risk of recurrence (2). RCC has a potential to metastasize to almost any site (3, 4) of which duodenum and pancreas are some of the rare locations for metastasis. Although the majority of metastases occur within 3 years of nephrectomy, it is a well-known fact that it can occur many years after nephrectomy. Most cases of duodenal metastasis from RCC present with upper gastrointestinal (GI) bleeding or obstructive symptoms (5). While pancreatic involvement is usually asymptomatic and usually detected on follow-up imaging for metastatic RCC, it may require the aid of esophagogastroduodenoscopy (EGD) to establish the diagnosis (6–8). Several therapeutic approaches have been documented including surgical and interventional therapy depending on the individual cases (5, 6, 9).

In recent years, with the advent of target therapies, the prognosis of patients with metastatic RCC has improved (10). However, in the absence of dramatic results, surgical excisions of isolated metastasis continue to play a role in the treatment of metastatic RCC (11–14).

## Materials and Methods

Within the last 10 years, six patients who were amenable to surgical excision presented to our service with metastatic disease to the duodenal/pancreatic area (Table 1).

**Table 1:** **Patient’s demographics**.

Patient	Sex	Age	Procedure	Time from initial surgery to metastasis (years)
1	M	75	Renal sparing surgery	19
2	M	58	R nephrectomy	12
3	F	66	R nephrectomy	4
4	F	21	L nephrectomy	2
5	F	52	L nephrectomy	1
6	F	70	L nephrectomy	10

This is a retrospective summary of the last 10 years where we treated six patients with duodenal/pancreatic metastasis following surgery for RCC. The demographics of these patients are presented on Table 1.

There were two males and four female patients varying in age from 21 to 75 years of age. Three patients had undergone L. radical nephrectomy, one a R. renal sparing surgery, and two R. radical nephrectomy.

Time of presentation of pancreatic metastasis varied from 1 to 19 years. In one patient, a distal pancreatic nodule was found and surgically excised at the time of nephrectomy. Twelve years later, the patient developed another metastasis to the head of the pancreas/duodenum.

On Table 2, we present the clinical/pathologic characteristic of the patients. Three patients had their metastasis discovered during routine follow up with CT scan, one patient presented with abdominal pain, and two patients with GI bleeding.

**Table 2:** **Clinical pathologic characteristics**.

Patient	Presenting symptoms	Site of metastasis	Surgical procedure	Pathology	Outcome
1	Routine FU	Distal pancreas	Distal pancreatectomy	*CCC	Alive
2	GI bleeding	Duodenal/head pancreas	Whipple	*CCC	Alive
3	Abdominal pain	Duodenal/head pancreas	Whipple	*CCC	Alive
4	Routine FU/CT	Distal pancreas	Distal pancreatectomy	**URCC	DOD
5	Retroperitoneal bleeding	Distal pancreas/spleen	Distal pancreatectomy	*CCC	DOD
6	Routine FU/CT	Distal pancreas	Distal pancreatectomy	*CCC	Alive

*Clear cell carcinoma; **Unclassified RCC.

The site of metastasis was the distal pancreas in four patients and the duodenal/head of the pancreas in two patients.

Four patients underwent distal pancreatectomy and two patients pancreatic duodenectomy (Whipple).

The pathology in four patients was a clear cell RCC, one an unclassified RCC, and one a clear cell carcinoma with rhabdoid characteristics.

Four patients are alive with periods varying from 1 to 8 years and two are dead of disease (DOD). Two patients underwent metastesectomy or cryotherapy for lung metastasis.

Two patients with aggressive tumors have succumbed to their disease despite several courses of target therapy.

## Discussion

Metastatic tumors of the small bowel are uncommon but certain tumors may metastasize more frequently than others, such as melanomas, lung cancer, cervical carcinomas, thyroid carcinomas, hepatoma, and Merkel cell carcinomas. RCC metastases represent 7.1% of these lesions and are most frequently located in the periampullary region or the duodenal bulb (3, 4, 10). Metastatic lesions to the pancreas are uncommon, accounting for only 5% of all pancreatic malignancies (7).

The natural history of RCC is unpredictable; the eradication of the disease is possible after nephrectomy, but there is a possibility of recurrence years after that. It has the potential to metastasize to almost any site but the most common sites are lung (75%), lymph nodes (36%), bone (20%), liver (18%), adrenal glands, kidney, brain, heart, spleen, intestine, and skin (2). RCC has been cited as the most common primary tumor leading to solitary pancreatic metastasis, representing 0.25–3% of all resected pancreatic specimens and the lesions can be multifocal in approximately 30% of patients and are resectable in approximately 80% of cases (7).

The routes of spreading can be peritoneal dissemination, hematogenous, lymphatic spread, and direct spread from an intra-abdominal malignancy (4). A majority of duodenal metastases, around 70%, occur from the right kidney due to the greater risk of regional invasion, as the patients we present (3, 6, 15).

The most common clinical presentation of GI metastases of RCC is GI bleeding, but it may present with abdominal pain, nausea, weight loss, early satiety, anemia and fatigue, jaundice, and duodenal obstruction or perforation (1, 16). On the other hand, pancreatic metastases could be asymptomatic in most cases and the early signs are often non-specific and subtle. Isolated pancreatic metastases are often found with routine surveillance imaging for primary lesions or as an incidental finding on imaging done for an unrelated indication. Others may present with abdominal pain (20%), GI bleeding due to duodenal infiltration (20%), obstructive jaundice (9%), weight loss (9%), pancreatitis due to pancreatic duct obstruction (3%), and pancreatic exocrine/endocrine dysfunction (7, 8, 17–19). In our experience, one patient presented with GI bleeding and another was asymptomatic, but his routine follow up allowed us to discover the recurrence of RCC to the pancreas.

Renal cell carcinoma is the primary tumor that metastasizes to the pancreas following the long-term disease-free interval, and the appearance of metastatic disease many years after nephrectomy is a well-known feature of RCC and is associated with a more favorable outcome than early recurrences, which tend to have a more rapid progression of disease (8), which is also seen in two of our patients (patients 4 and 5), who developed an early metastasis from the primary resection and died because of that.

The approach to the management of patients with those symptoms, and a history of RCC should include a full diagnostic work-up with radiologic evaluation with a computer tomography (CT) scan with contrast and/or endoscopic evaluation. In case of duodenal compromise, the CT scan may show thickening of the wall or folds in the involved segment and the lesion in the upper endoscopy can be seen as a submucosal mass with ulceration of the tip, multiple nodules of different sizes, and raised plaques (3–6). In case of pancreatic metastasis, the CT scan shows hyper enhancing lesions that are most pronounced in the early arterial phases of enhancement, which reflects the hyper vascular nature of these tumors (19). The most common type RCC metastasis, reported in 50–73% of cases, is that of a solitary, localized, well-defined mass. A second pattern of multiple pancreatic lesions has been reported in 5–10% of cases and a third pattern of diffuse metastatic infiltration causing generalized enlargement of the organ in 15–44% of cases (20). In our patients, we also appreciate that the most common pattern of metastasis is a single mass, either in the head of pancreas or the tail of the pancreas. Although the final diagnosis is made by biopsy with fine-needle aspiration, we should take into account the risk of bleeding, which is increased due to the high vascularity of the tumor. We did not take a biopsy to any patients before the surgery due to the high risk of bleeding.

Currently, management depends on the general condition of the patient, the extent and location of the lesion, and the concurrent metastases at other sites. In the case of duodenum metastasis procedures from classic pancreaticoduodenectomy (Whipple procedure) to interventional embolization have been reported (1–6). We only use the surgical procedure due to the self-limiting bleeding in one of our patients.

Any patient with solitary metastatic RCC to the duodenum should be a candidate for complete surgical excision if medically and technically feasible, both for palliation and prognostic reasons, as it appears to prolong disease-free survival. This kind of surgical resection has resulted in 5-year survival rates from 35 to 50% and 5-year disease-free survival rate of 5 to 23% (1). But for people with disseminated malignancy, the average survival is about 4 months and only 10% of these survive for 1 year, and the treatment is mainly supportive and palliative (3, 4).

If the patient developed pancreatic metastasis, a standardized pancreatic resection is used and this is tailored according to the location of the tumor, including partial pancreaticoduodenectomy, distal pancreatectomy, and total pancreatectomy for the management of isolated pancreatic metastasis, which can be made either laparoscopically or through using transumbilical single incision (8, 21). Five-year survival ranges from 29 to 35% and some studies reports up to 81%; however, multiple metastatic lesions carries worse prognosis than solitary ones (7, 22).

Recently, a review of the role of pancreatic metastatic RCC, from an Italian Center, suggested that pancreatic metastasis was associated with better prognosis (23). The authors reviewed records of 354 patients with metastatic RCC, 3 with synchronous, and 11 with metachronous metastasis. Surgical excision was performed in two patients. Median overall survival was 39 months for patients with pancreatic metastasis as compared to 23 months for metastatic disease elsewhere (23).

The advent of target therapy for metastatic RCC has increased the median tumor-specific survival for those patients (10). The complete resection of metastasis when found in a single organ, has considerable impact on survival (11–14).

Because, we cannot closely monitor the patients in this paper, we can neither quantify the importance of monitoring them, nor determine for how long should be monitored for metastasis; further studies are needed to address this issue.

## Conclusion

Renal cell carcinoma metastasis to duodenum and pancreas is a rare occurrence; however, with this report, we can intuit that it is prudent to investigate the presence of any GI symptoms in these patients to rule out the presence of tumor recurrence and to consider resection as the procedure of choice whenever possible.

## Conflict of Interest Statement

The authors declare that the research was conducted in the absence of any commercial or financial relationships that could be construed as a potential conflict of interest.
